# Patient Perspectives of Skeletal Muscle Cramping in Dialysis: A Focus Group Study

**DOI:** 10.34067/KID.0000000000000121

**Published:** 2023-04-08

**Authors:** Amanda Grandinetti, Tandrea S. Hilliard-Boone, Kenneth R. Wilund, Dilani Logan, Wendy L. St Peter, Rebecca Wingard, Francesca Tentori, San Keller, Melissa West, Eduardo Lacson Jr, Michelle M. Richardson

**Affiliations:** 1 Kidney Health Initiative Patient and Family Partnership Council, Washington, District of Columbia; 2 American Institutes for Research, Health, Chapel Hill, North Carolina; 3 Department of Kinesiology and Community Health, University of Illinois at Urbana-Champaign, Champaign, Illinois; 4 American Institutes for Research, Health, Oakland, California; 5 College of Pharmacy University of Minnesota, Minneapolis, Minnesota; 6 Fresenius Medical Care North America, Clinical Services, Waltham, Massachusetts; 7 Davita Clinical Research, Minneapolis, Minnesota; 8 American Society of Nephrology, Washington, District of Columbia; 9 Dialysis Clinic, Inc., Nashville, Tennessee; 10 Tufts Medical Center, Boston, Massachusetts

**Keywords:** dialysis, ESKD, muscle cramping, focus group interviews, qualitative research

## Abstract

**Key Points:**

- This first step demonstrated content validity for a patient-reported outcome measure for skeletal muscle cramping in dialysis.
- This work lays the foundation for developing a patient-reported outcome measure for regulatory use to assess skeletal muscle cramping in people receiving dialysis.

**Background:**

Skeletal muscle cramping is a common, painful, and debilitating symptom experienced by people receiving dialysis. Neither a standardized, patient-endorsed definition of skeletal muscle cramping nor full understanding of patients' perspectives of skeletal muscle cramping exist. We conducted focus groups, within a Kidney Health Initiative (KHI) project, to elicit skeletal muscle cramping experiences of people receiving dialysis as the basis for patient-reported outcome measure (PROM) development.

**Methods:**

Eligible participants (English-speaking adults aged 18–85 years treated by dialysis and a skeletal muscle cramping episode within 30 days) were purposively recruited from a panel (L&E Research) of people receiving dialysis at home or in-center. Standard qualitative methods were used to conduct virtual 90-minute sessions discussing the following: skeletal muscle cramping clinical characteristics, participants' skeletal muscle cramping experiences, and feedback on a draft skeletal muscle cramping definition and a patient-facing conceptual model developed by the KHI project workgroup. We used qualitative thematic analysis.

**Results:**

There were 20 diverse participants in three focus groups. Universally experienced skeletal muscle cramping attributes differed by dialysis setting in onset, worst pain rating, duration, and timing. Variably experienced attributes (applied to home and in-center dialysis) were gross and fine motor effect, sleep disruption, mood-related themes of fear, and annoyance/frustration/irritability. Avoidance/adaptive behaviors included reluctance or avoiding movement, adjusting what they ate or drink (*e.g.*, yellow mustard, pickles, pickle juice, and tonic water), heat application, massage, and cannabidiol use. The skeletal muscle cramping definition was endorsed, and insightful suggestions for conceptual model were collected.

**Conclusions:**

This qualitative study of in-center and home patients' skeletal muscle cramping experiences identified universally and variably experienced attributes. The patient-endorsed skeletal muscle cramping definition can serve as a standard for assessment. These results provide the foundation to develop a PROM for regulatory use with people receiving maintenance dialysis who experience skeletal muscle cramping.

## Graphical abstract

**Figure:**
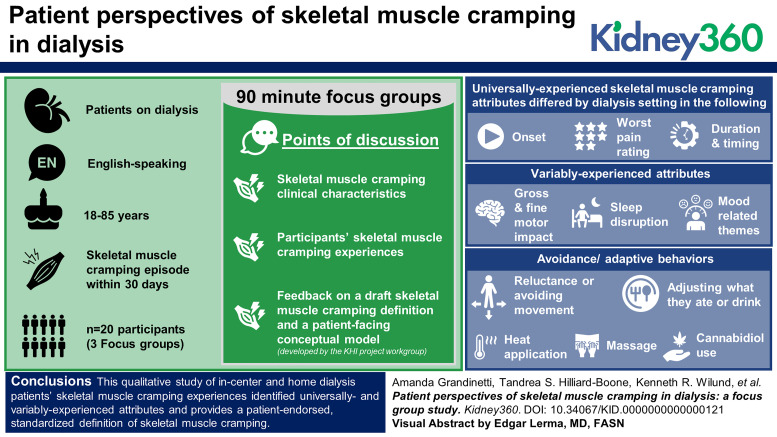

## Introduction

An urgent need exists for both therapeutic innovations in kidney failure and a paradigm shift to focus on patients' priorities and values.^1–6^ Patient-centric approaches are essential because people receiving dialysis have poor quality of life, adverse effects from treatment, and high symptom burden.^7–11^ Skeletal muscle cramping, for example, is a common, painful, and debilitating symptom experienced by people treated with dialysis, regardless of modality.^12–15^ The estimated prevalence of skeletal muscle cramping varies widely, likely because there is neither a standardized definition nor consistent measurement approach. People receiving dialysis have ranked skeletal muscle cramping as a priority for innovation because meaningful improvement in this symptom would improve overall quality of life.^16^

A standardized, patient-endorsed skeletal muscle cramping definition and full understanding of patients' perspectives are needed to achieve this priority and advance therapeutic innovation to prevent and/or treat skeletal muscle cramping. Symptoms, such as skeletal muscle cramping, cannot be assessed using biomarkers and must be measured using patient-reported outcome measures (PROMs) that query the individual directly and avoid clinician judgment or interpretation. Irrespective of disease state, few, if any, PROMs capture the entirety of skeletal muscle cramping clinical characteristics (*i.e.*, onset, frequency, location, severity, duration, timing, and cause). There are two dialysis-related PROMs that assess skeletal muscle cramping, both of which have limitations. The Kidney Disease Quality of Life-36 survey has one question assessing the amount of bother from skeletal muscle cramping,^17^ and a study-specific PROM^18^ has unknown psychometric characteristics. Neither comprehensively assesses skeletal muscle cramping in people receiving maintenance dialysis.

Given these factors, the Kidney Health Initiative (KHI, www.kidneyhealthinitiative.org), a private–public partnership between the American Society of Nephrology (ASN), US Food and Drug Administration, and over 100 members whose mission is to catalyze innovation and the development of safe and effective patient-centered therapies for people living with kidney disease,^1^ convened a multidisciplinary workgroup inclusive of patients, clinicians, and measurement experts to consider skeletal muscle cramping as a continuation of its former work on symptoms of patients on dialysis. In the previous activity, muscle cramping was prioritized by patients receiving dialysis as one of the top three symptoms for which they would like relief.^16,19^ The goal of the patient-reported outcomes for muscle cramping in patients on dialysis workgroup was to define a set of PROMs that could be used in future clinical trials to test the effect of new dialysis devices, new kidney replacement therapy technologies, lifestyle/behavioral modifications, and medications on alleviating skeletal muscle cramping. After a literature review identified no applicable PROMs and a general lack of information on skeletal muscle cramping experiences in dialysis,^20^ ASN partnered with the American Institutes for Research (AIR) to conduct a focus group study to broadly elicit the skeletal muscle cramping experiences of people receiving dialysis to inform PROM development. The methods and results for the complete workgroup's product are published elsewhere.^20^

## Materials and Methods

### Focus Group Participant Selection, Setting, and Data Collection

We conducted three virtual focus groups (two for people receiving dialysis in-center and one for people receiving dialysis at home, either peritoneal dialysis or home hemodialysis). Eligible participants (*1*) were English-speaking adults aged 18–85 years, (*2*) had kidney failure currently being treated by dialysis, and (*3*) experienced skeletal muscle cramping in the last month. Participants received $75 for participating in the 90-minute focus group. This project was approved by the AIR Institutional Review Board (IRB00000436/FWA00003952) with waiver of informed consent.

Owing to the coronavirus disease 2019 pandemic and travel restrictions, L&E Research (www.leresearch.com), a recruitment firm, identified participants from their large, nationwide panel of engaged and diverse people with CKD, including those receiving dialysis. The workgroup developed a list of desired characteristics on which we sought diversity. AIR provided L&E with eligibility criteria, the list of desired characteristics, and a recruitment screener. L&E applied a purposive recruitment strategy to identify eligible individuals on the panel representing diverse backgrounds, including sex, race, ethnicity, education, geographic location, income range, and time on dialysis. L&E conducted telephone outreach to fill three focus groups with 6–8 participants each. By design, one group consisted of participants currently treated with home dialysis because their skeletal muscle cramping experiences are underrepresented in the literature. Also by design, we solely focused on input from people with CKD treated with dialysis and not from health professionals. Systematically, large-scale, international research has shown that people with CKD consistently give higher priority to symptoms and life effects than do health professionals.^5,6^ Furthermore, we placed highest priority on the person's experience of skeletal muscle cramping to inform our goal of developing a PROM to assess the effect of new dialysis devices, new kidney replacement therapy technologies, lifestyle/behavioral modifications, and medications on alleviating skeletal muscle cramping.

We prepared tailored moderator's guides (Supplemental Material 1) using results from the literature review^20^ and workgroup member recommendations. Topics included skeletal muscle cramping clinical characteristics (*i.e.*, onset, frequency, location, severity, duration, timing, and cause), participants' skeletal muscle cramping experiences (*i.e.*, skeletal muscle cramping descriptions, amount of bother, interference and life effect, treatment effect, communication with providers, and remedies used), and feedback on a skeletal muscle cramping definition and a patient-facing conceptual framework.

**Figure 1:**
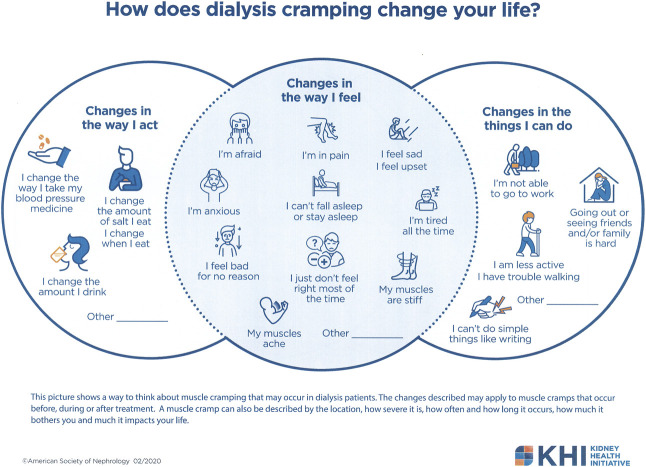
**Patient-facing conceptual framework.** The patient-facing conceptual framework was adapted from the initial framework developed by workgroup members to guide the project^20^ with the intent of using it during the focus groups. The organization of the patient-facing framework was different from the initial framework which was organized by precramping, during, or postcramping episode. The patient-facing framework included the same concepts but was organized around changes in the way patients may act, feel, or do because of skeletal muscle cramping.

Receiving patient feedback and endorsement on a skeletal muscle cramping definition and the patient-facing conceptual model was a primary motivation for conducting the focus groups. The proposed definition of skeletal muscle cramping in dialysis provided to participants was “*Muscle cramps are involuntary painful skeletal muscle contractions anywhere on the body, occurring during or between dialysis treatments, day or night.”* A graphic artist converted the workgroup's initial hypothesized conceptual framework,^20^ developed to organize our thoughts, and represent a path forward, into an easily understandable version (Figure 1) with three main areas—changes in: (*1*) “Things I Can Do,” (*2*) “Way I Feel,” and (*3*) “Way I Act.”

Participants joined using the virtual conferencing software Go-To-Meeting (https://www.gotomeeting.com/) with screen sharing of materials or dialed in through telephone, if they preferred. L&E Research emailed (or mailed if needed) background information and the patient-facing conceptual framework to confirmed participants in advance of the meetings. An experienced female moderator from AIR (author T.S.H.-B.), who had no previous contact with focus group participants, led all sessions with support from an AIR note taker (author D.L.) and ASN staff observer. This same moderator conducted the focus groups for previous KHI workgroups.^16,19^ Sessions were audio recorded and professionally transcribed and documented. Participant characteristics were self-reported.

### Qualitative Data Analysis

Immediately after each session, AIR staff debriefed to share their initial impressions and understanding and referenced the audio recording and detailed meeting notes, as needed, to ensure that all important dialog and context were accurately captured. The first focus group provided unanticipated feedback on the patient-facing framework. We then revised the moderator's guide for the subsequent groups to incorporate probes to elicit the rationale for dissenting opinions of the patient-facing framework.^21^

Meeting transcripts were reviewed and systematically coded using NVivo 12 software. Two analysts collaboratively coded the data using a central codebook, including deductive codes, on the basis of the focus group interview protocol. The codebook captured concepts related to (*1*) patients' experiences with muscle cramping (terms to describe muscle cramping, timing of muscle cramps in proximity to dialysis treatment, frequency, location, causes, duration, pain severity, amount of bother, pain interference and life effects, treatment effect, communication with providers, and remedies), (*2*) patients' agreement with or recommended changes to the proposed definition of muscle cramping, and (*3*) patients' feedback on the conceptual framework (general reactions and recommended changes and prioritization of model components). Analysts applied these deductive codes and debriefed to resolve discrepancies and revise the initial code list as appropriate. Conclusions were drawn by identifying and interpreting patterns, themes, and subthemes both within and across groups. Workgroup members also reviewed the focus group transcripts and transcript coding findings. We used the Consolidated Criteria for Reporting Qualitative Research^22^ to report this study.

## Results

### Timing and Demographics

We conducted focus groups in August and September 2020. A total of 24 participants were recruited (eight in each group) and 20 participated (13 in-center dialysis, seven home dialysis—three home hemodialysis and four peritoneal dialysis). Four people confirmed attendance but did not participate (three no shows and one withdrew for a medical procedure). Nearly all participants chose to attend using computer versus joining in over the telephone. In each focus group, participants were engaged and participatory. Any minor technical complications that occurred were easily overcome and did not affect the focus group interactions. Table 1 presents participant demographics by the focus group.

**Table 1:** Focus group participants' demographic characteristics

Characteristic	Focus Group Participants (*N*=20)		
	Focus Group 1: In-Center Hemodialysis (*n*=6)	Focus Group 2: In-Center Hemodialysis (*n*=7)	Focus Group 3: Home Hemodialysis or Peritoneal Dialysis (*n*=7)
	*n* (%)		
**Age, yr**			
20–29	0 (0)	0 (0)	2 (29)
30–39	1 (17)	1 (14)	1 (14)
40–49	2 (33)	1 (14)	1 (14)
50–59	2 (33)	4 (57)	2 (29)
60–69	1 (17)	1 (14)	1 (14)
**Sex**			
Male	4 (67)	3 (43)	3 (43)
Female	2 (33)	4 (57)	4 (57)
**Education level**			
Less than high school	0 (0)	0 (0)	0 (0)
High school graduate	1 (17)	1 (14)	0 (0)
Technical/vocational training	0 (0)	1 (14)	0 (0)
Associates degree	2 (33)	1 (14)	1 (14)
Some college	2 (33)	4 (57)	5 (72)
College graduate	1 (17)	0 (0)	1 (14)
**Race/ethnicity**			
Black	2 (33)	3 (43)	5 (72)
White	3 (50)	3 (43)	1 (14)
Hispanic/Latino	0 (0)	0 (0)	1 (14)
Asian/Pacific Islander	0 (0)	0 (0)	0 (0)
American Indian or Alaska Native	0 (0)	0 (0)	0 (0)
Other	1 (17)	1 (14)	0 (0)
**Time on dialysis**			
6–11 mo	0 (0)	0 (0)	1 (14)
1–5 yr	5 (83)	5 (71)	3 (43)
6–10 yr	0 (0)	1 (14)	1 (14)
>10 yr	1 (17)	1 (14)	2 (29)
**Comorbidities**			
Diabetes	2 (33)	4 (57)	3 (43)
Hypertension	4 (67)	6 (86)	5 (71)
Cardiovascular disease	3 (50)	3 (43)	2 (29)
Congestive heart failure	0 (0)	0 (0)	0 (0)
Lung problems	0 (0)	2 (29)	1 (14)
Peripheral vascular disease	0 (0)	2 (29)	2 (29)
Neurological	1 (17)	3 (43)	3 (43)
Vision impairment	1 (17)	1 (14)	2 (29)
Other/unspecified	3 (50)	2 (29)	2 (29)

### Skeletal Muscle Cramping Clinical Characteristics

All groups explicitly conveyed that skeletal muscle cramping clinical characteristics (*e.g.*, severity, frequency, and location) did not represent the entirety of their skeletal muscle cramping experiences. After data were coded and analyzed, we categorized participants' experiences of skeletal muscle cramping into categories of universally and variably experienced attributes. Universally experienced attributes were those characteristics that would apply to and able to be collected for all episodes of skeletal muscle cramping (*i.e.*, severity, frequency, location, duration, and timing). Variably experienced attributes represent characteristics that would not apply in all situations of skeletal muscle cramping (*e.g.*, effects on fine motor control would only be in cases of skeletal muscle cramping in the hands or wrist and not all patients have that location for skeletal muscle cramping). All themes and subthemes apply regardless of home or in-center dialysis. Table 2 presents illustrative participant quotes relating to identified running themes and subthemes.

**Table 2:** Illustrative quotations of participant perspectives on skeletal muscle cramping

Concepts	Patient Quotes
Pain—acute	“Mine has been so severe, it has made me sick on the stomach. That's how bad I've had some of them.” (home) “You know, for me, that's bad, because I know ten is the worst pain that you've experienced, but there's time when you go—if there was something that came after 10, that's where it's at.”(home) “These are real painful cramps, too, because I've worked out all my life. I've cramped up for lack of fluid in my younger days, too, but these can feel like it's pulling the muscle from the bone.” (in-center)
Pain—lingering	“The only time I've noticed continuing pain is when the cramps are in my hands, and it's only happened once or twice, that once my hands finally relax, my fingers hurt from being clenched in a weird position for so long. ”(in-center) “…If it's gotten to the point where it's serious, then there will be some awkwardness in moving afterward. You know, for me, it's usually in my feet and legs, and it might be the next day, and I'm like, hey, how come I have soreness in that area? Oh, yeah, I had the cramp. And then I will maybe have that weakness that you're talking about and not really be able to ambulate efficiently.” (in-center)
Gross motor	“…And I notice that for some reason my calves lately, when you stretch, I can't do that, because if I stretch, the cramp intensifies, and sometimes I've got to jump off the bed to kind of walk it out.” (home) “You don't want to exert yourself.” (in-center) “At dialysis, I refrain from stretching, because that brings on cramps, a lot of times.” (in-center) “I find that exercising can be kind of a Catch-22. If you don't exercise, you kind of lose muscle tone, and I feel like I get cramps easier. But when I exercise, I tend to bring cramps on because of the lack of fluid.” (in-center)
Fine motor	“Yeah, on my right hand, two of my fingers have been locking up for I want to say the last couple of months. Some days it's better than other days, but just to hold a cup can be aggravating.” (home) “I'm a gamer, and - well, I used to game. Ever since I started dialysis, I kind of stopped gaming, because my hands are going to cramp up.” (home)
Sleep	“Mine has affected my sleep. I'm subject to have to jump up out of the bed, either try to walk it out or just try to get comfortable all over again, and sometimes it's hard once you get woke up out of a good sleep.” (home) “Well, it affects my sleep, but also it affects me during the day, because you're wondering if you do too much with that particular muscle or my hand, is that going to make you hurt worse that night? And so sometimes I really limit how much I'm going to do. Hopefully that's going to limit how bad it's going to hurt.” “They don't, really, other than interrupting sleep every once in a while.” (in-center) “I usually feel tired. When I get home, I'm ready to go to bed. I'll lay down and I'll fall asleep for a few hours. I'm exhausted. And even the next day, I usually feel tired also.” (in-center)
Fear	“Yes, because they feel they're never going to stop.” (in-center) “I'm scared because I find it very painful. Like I'm going to wonder, when is it going to light up?” (in-center)
Mood (irritability, frustration, aggravation)	“She definitely can tell when I'm irritated, and I have to apologize to her, like ‘Mom, look, I'm not mad at you, I'm just in pain. I'm trying to figure this out.’ So dealing with family and friends, I try not to be around too many people because I know I can turn into a sour patch kid real fast.” (home) “One word would be annoying, because it stops me from doing things that I want to do at times.” (home) “Yeah, I almost feel like I'm in a better mood when they're over than when they started, because I'm like, ah, thank god that's done.” (in-center) “So at that moment, when you're having those cramps, you're giving these very short answers and anxious, anxiety—all of it, you know. But when it's over with, it's fine. It's normal.” (in-center)
Adaptive behaviors	“It's hurt, at time, enough that I've had to stop treatment” (home) “…If you're talking about preventative, if you're taking anti-inflammatory things, antioxidant-rich food and supplements, that would be another approach, I believe.” (in-center) “I've come off my machine early so that I could move around, because we couldn't get it to stop.” (in-center) “…Because, like I said, for my treatment, if I drink a lot, then it's pulled more, and I'm most likely going to cramp. So I do not want that. So I try to monitor my liquid intake.” (in-center)
Use of remedies	“Mostly off guard. If I feel it coming, I try and do something preemptively, like the heating pad, and massage my hand, but I do it to satisfy my mind, but it does not make a difference” (home) “I use the CBD, but also, I use cyclobenzaprine.” (home) “I keep mustard beside my bed...Actually you take it. I Take a teaspoonful of it.” (home) “…also, pickle juice.” (in-center) Long-term, magnesium has helped me get not as many cramps. That was something my nephrologist recommended.” (in-center) “If I have a really terrible cramp that won't go away, at my center they give you bouillon, like a little bit of powdered bouillon” (in-center)

### Participants' Skeletal Muscle Cramping Experiences

#### Universally Experienced Skeletal Muscle Cramping Attributes

Table 3 presents the universally experienced skeletal muscle cramping attributes by home and in-center dialysis treatment locations. These attributes comprise skeletal muscle cramping clinical characteristics.

**Table 3:** Skeletal muscle cramping clinical characteristics by dialysis treatment location

Characteristic	Home	In-Center
Onset	Caught off guard	Caught off guard^a^
Location^b^	Lower extremities—thighs, calves, feet. Hands, fingers, and torso/ribs	Lower extremities—thighs, calves, feet Hands, fingers, and torso/ribs
Most severe pain rating (one no pain–ten worst)	>10	8
Average pain rating (one no pain–ten worst)	4–6	2–10
Proximity to treatment and time of day	During and outside of dialysis and at night	During dialysis
Duration^c^	Less than 10 min	5–10 min but some as long as 15–20 min
Participant-identified causes	Taking off too much fluid during dialysis Wrong movements	Taking off too much fluid or too quickly during dialysis

a Being caught off guard was particularly evident in the in-center focus groups.

b All locations were reported in all focus groups. However, most participants reported lower extremity skeletal muscle cramp location.

c A few patients in each focus group reported experiencing longer-lasting skeletal muscle cramps.

The primary universal attribute identified in all groups was acute pain at the site of the skeletal muscle cramp. Many in-center participants stated that their skeletal muscle cramping was very painful. A few participants described wanting to jump up, scream, or cry when the skeletal muscle cramp started, even while receiving dialysis. Home participants described skeletal muscle cramping as painful and causing them to feel as if they were sick or pulled a muscle. A pain-related subtheme was lingering pain described as soreness, stiffness, or tense muscles. More than half of home group participants and a few in-center participants reported soreness and stiffness in the area of the skeletal muscle cramp.

Notable differences in clinical characteristics by dialysis setting include the following: a few home participants, but nearly half of the in-center participants reported being caught off guard by skeletal muscle cramping; the most severe pain rating for home participants exceeded the maximum of ten versus a maximum of eight for the in-center group; skeletal muscle cramp duration was <10 minutes for home participants, but some in-center participants described skeletal muscle cramping lasting as long as 15–20 minutes; and timing of skeletal muscle cramping occurred during and outside of dialysis, especially at night for home participants, but in-center participants described skeletal muscle cramping occurring primarily during dialysis, typically occurring in the middle of treatment.

#### Variably Experienced Skeletal Muscle Cramping Attributes

Variably experienced skeletal muscle cramping attributes only apply in certain circumstances and in those cases are important perspectives. However, they likely do not apply to all patients or in all circumstances.

#### Physical Function

Across all groups, most participants reported skeletal muscle cramping in their lower extremities. Lower extremity skeletal muscle cramps resulted in gross motor effects, such as inability to move, awkwardness in movement, and for a few in-center participants, it created challenges for staying active and exercising. Although skeletal muscle cramping in upper extremities (forearm, hands, and fingers) was less common, those who experienced it reported challenges with everyday tasks (*e.g.*, cooking, holding or picking up items, and driving). Fine motor effect also created challenges for leisure activities (*e.g.*, playing video games or fishing).

#### Sleep

Participants reported several characteristics of nocturnal skeletal muscle cramping associated with sleep, including sleep interruption, drowsiness, and tiredness. Participants receiving dialysis in-center reported being particularly tired after a dialysis session in which skeletal muscle cramping occurred.

#### Mood

The two predominant mood-related themes were fear and annoyance/frustration/irritability. Fear was a predominant subtheme described in the in-center groups and included both fear of the skeletal muscle cramp itself and fear that subsequent movement may precipitate skeletal muscle cramping. All groups described annoyance/frustration/irritability as occurring mostly during skeletal muscle cramping which manifested in their interactions with caregivers or family members (home participants) and dialysis staff (in-center participants). Anxiety was only mentioned in one in-center group and was not a theme.

#### Avoidance/Adaptive Behaviors

Participants reported many avoidance or adaptive behaviors to prevent or minimize skeletal muscle cramping. All groups discussed remedies or treatments they used to relieve or prevent skeletal muscle cramping, including adjusting what they eat or drink, *e.g.*, yellow mustard, pickles, pickle juice, and tonic water; holding a hot water balloon or using a heating pad; taking magnesium; anti-inflammatory medications and antioxidant-rich foods; using a leg massager; and using cannabidiol. Home participants did not report dietary or fluid intake changes. In-center participants discussed diet and fluid changes, especially modifying fluid intake, but none mentioned dietary salt intake or noted the association of high sodium-containing remedies with potentially increasing fluid intake. Areas that the workgroup identified before the focus group sessions on the basis of clinical experience (*e.g.*, stopping or shortening treatments) were briefly discussed but were not as predominant. Participants with lower extremity skeletal muscle cramping were reluctant to move and avoided or limited movement, stretching, or exercise, particularly during but not necessarily limited to the immediate postcramp period.

### Focus Group Feedback on Definition of Skeletal Muscle Cramping

Most focus group participants agreed with the proposed skeletal muscle cramping definition. Suggestions included explicitly using words, such as annoying, aggravating, intense, and painful; skeletal muscle cramping could occur anytime or anywhere; skeletal muscle cramping was part of dialysis or their reality; and skeletal muscle cramping is one of the most challenging parts of dialysis. We then revised the definition to *“Muscle cramping that maintenance dialysis patients experience are involuntary, painful, sometimes intense, skeletal muscle contractions anywhere on the body, occurring at any time, day or night.”* We chose not to include the suggested components (annoying, aggravating, part of reality, and most challenging part of dialysis) as those characteristics may not be universally experienced.

### Focus Group Feedback on the Patient-Facing Conceptual Framework

Feedback on the framework (Figure 1) was mixed. Participants noted that the framework included items not related to their skeletal muscle cramping experience (*e.g.*, difficulty going out or seeing friends and/or family and changing fluid intake or blood pressure medicine). One in-center group stated that the framework represented the entire dialysis experience and was not specific to skeletal muscle cramping. A few participants thought that the framework would be helpful for people new to dialysis treatment. However, one participant stated that they would have been afraid and anxious if they had received the framework at that time. Home participants had positive feedback. Although some acknowledged that all the points may not be relevant to each individual, they liked the format. Participants recommended changes, such as making each topic a full circle and because some topics may not be relevant to everyone, the groups recommended modifying the framework so that it could be customized by patients and providers to reflect their own experiences. A few participants suggested that acknowledging skeletal muscle cramping effects were temporary and that reactions to skeletal muscle cramping and the presence of skeletal muscle cramping itself changes over time.

## Discussion

This is the first qualitative study to assess both in-center and home dialysis settings and peoples’ experiences of skeletal muscle cramping in those locations. We categorized skeletal muscle cramping attributes into universally experienced (skeletal muscle cramping characteristics, including pain) and variably experienced (gross and fine motor, sleep, and mood) attributes that will contribute to PROM development. We also elucidated the wide-ranging behaviors that people receiving dialysis use to avoid or adapt to skeletal muscle cramping. We created a standardized, patient-endorsed definition of skeletal muscle cramping and patient-facing conceptual framework that, although not aligned with our original intent, could be an education tool applicable to the dialysis experience. These results represent the first step in demonstrating content validity^23^ to support developing a comprehensive PROM for people receiving dialysis and experiencing skeletal muscle cramping.

In addition, we discovered data that could inform clinical care, although not the main focus of this work. In-center, but not home participants, reported dietary and fluid intake changes because of skeletal muscle cramping. This difference in patient experience may be due to longer home dialysis treatment times with lower ultrafiltration rates along with less restrictive diet and fluid intake with home dialysis therapies. In-center participants reported modifying fluid intake, but none mentioned the role of sodium intake as important for managing fluid intake although some of the remedies they reported are high in sodium. People receiving dialysis and experiencing skeletal muscle cramping may benefit from education to better understand the role of high sodium intake to increase thirst and subsequent fluid intake, complicating proper fluid management. Stopping or shortening treatments as avoidance/adaptive behaviors were reported by some patients but was not as prevalent or deemed as important as other effects of skeletal muscle cramping. Furthermore, mood effects of skeletal muscle cramping could alter relationships between caregivers or clinic staff with patients and then affect the outcomes of clinical care. In some instances, participants stated that dialysis staff did not care about them during skeletal muscle cramping episodes.

Several participant comments were compelling, hypothesis generating, and could inform future research and innovations to prevent or treat skeletal muscle cramping and suggest that innovation may have effect beyond skeletal muscle cramping itself. For example, the fear of movement or inability to move after skeletal muscle cramping has not previously been elucidated. Perhaps skeletal muscle cramping is related to the relative inactivity of some people receiving dialysis and/or prevents or limits their engagement with recommendations for physical activity. In addition, themes of inactivity or fear of movement, tiredness and sleep disruptions, or lingering pain from hand cramps may contribute to the inability to prepare healthy food, poor nutritional status, or the reliance on convenience foods in some people receiving dialysis. More research is needed to examine how skeletal muscle cramping affects physical activity and nutrition behaviors.

Our study has many strengths. We used well-accepted methods and a highly experienced focus group facilitator to minimize observation bias and optimize participant interaction and participation in a virtual setting. The purposive recruitment strategy resulted in the desired diverse group of participants, and we included participants receiving home dialysis, which have not been included in previous work. The focus groups allowed us to critically examine the real-life experiences of skeletal muscle cramping in people receiving dialysis.

There are limitations to this research, and opportunities exist to confirm these findings. Our participants had, on average, more years of educational attainment than the general dialysis population in the United States. Although sample size is small, it is consistent with similar studies, and we expect that other qualitative analyses would yield similar results because many of the themes have been reported in previous evaluations of skeletal muscle cramping in other disease states and are consistent with clinical observations. The home dialysis focus group consisted of patients receiving peritoneal dialysis or home hemodialysis, so we are not able to make conclusions about differences in home modalities within the home dialysis group. However, engagement was consistently strong both within and among focus groups. Our predetermined focus on the patient and their experience of skeletal muscle cramping limited our participants to only patients and perspectives of health professionals were not elicited.

A gap exists in understanding of skeletal muscle cramping duration. We are only able to report broad time intervals that participants offered. Additional work is needed to evaluate skeletal muscle cramping duration, and when integrated into a PROM for regulatory use, response options will need to be provided in time intervals that are meaningful to patients. On the basis of our results, consideration should be given to recording duration >10 minutes as a few participants reported longer skeletal muscle cramping episodes.

This work lays the foundation for developing a PROM for regulatory use to assess skeletal muscle cramping in people receiving dialysis. The workgroup final report^20^ provided a conceptual framework that is the basis for the next steps. A comprehensive PROM needs to be developed using accepted research methods and the themes we elicited to systematically measure the patient experience of skeletal muscle cramping. At a minimum, we propose that PROMs for skeletal muscle cramping in dialysis include patient-reported clinical characteristics (onset, frequency, location, severity, duration, and timing in relation to dialysis treatment) and the universally experienced attribute of pain. Although these are characteristics that clinicians traditionally evaluate, there is a need for a reliable and valid PROM for people on dialysis that allows for systematic and reproducible patient reporting that minimizes or avoids clinician judgment or interpretation. This is especially important as new devices to assist people on dialysis are in development. A PROM for regulatory use could be valuable in the medical device clinical trials. Furthermore, there may be a role for assessing the variably experienced attributes depending on study purpose or the innovation being assessed. Systematically evaluating skeletal muscle cramping in people treated with dialysis using a reliable and valid PROM will allow patients, clinicians, researchers, and stakeholders the opportunity to prioritize patients' experiences, better understand the effect of skeletal muscle cramping on patients' lives, evaluate new treatments and approaches, and improve clinical care.

Incorporating the patient experience is one of the most important aspects of developing new therapies and driving innovation. In particular, PROMs should be developed in partnership with patients from beginning to end to ensure they are patient-centered and represent their priorities. Patients are more likely to adopt treatment when they know how a drug or device will improve their quality of life and achieve their goals. We posit that this work successfully incorporated the patient perspective and completed the necessary initial step toward standardized, systematic evaluation of skeletal muscle cramping in the maintenance dialysis population.

## Supplementary Material

SUPPLEMENTARY MATERIAL

## Data Availability

All data is included in the manuscript and/or supporting information.

## Supplemental Material

Supplemental Material 1. Focus group questions.
