# Supplementary material for: Patient Perspectives of Skeletal Muscle Cramping in Dialysis: A Focus Group Study
Source: Kidney360. 2023 Apr 8;4(6):e734–43. doi: 10.34067/KID.0000000000000121 (PMC10371365; doi:10.34067/KID.0000000000000121)
Supplement: SUPPLEMENTARY MATERIAL [file kidney360-4-e734-s001.pdf]

Supplemental Material 1: Focus Group Questions

Patients' Muscle Cramping Experiences

Recent experiences with cramping

- Think of one word that describes the muscle cramps you've had in the past month.
- What word did you think of and why?

Time and proximity to treatment

- When you think about your day, when do the cramps usually happen?
  - [Applies to in-center HD and home HD patients only] Is this different for dialysis and non-dialysis days?
  - Do the cramps tend to happen before, during, or after dialysis treatment?
- Do they happen mostly during the day when you're active, at night when you're resting, or any time?

Frequency

- How often do the muscle cramps occur before dialysis? During dialysis? After dialysis?
  - Do you have more than one muscle cramp during a dialysis session?
- Have you experienced more, less, or the same muscle cramps in the past month compared to before the COVID-19 pandemic?
- Do you anticipate (expect) the muscle cramps or do they tend to come unexpectedly (catch you off guard)?
- Do you fear getting muscle cramps?
- Have you been more fearful or anxious about getting muscle cramps during the COVID-19 crisis than you were before?

Location

- Where in the body do you experience cramps?
  - Do cramps tend to happen more often in certain parts of your body than others?
  - Do cramps come one after the other in the same place or do you get multiple cramps in different parts of your body at the same time?

Causes

- What do you think causes the muscle cramps you experience?
- Do you think the muscle cramps you get are because of the dialysis treatment? From missing/shortening dialysis treatments?

Duration

- How long do your muscle cramps last?
  - Do they tend to last longer in some parts of the body versus others?

Pain Severity and other Effects

- What do the muscle cramps feel like?
- How do the muscle cramps make your body feel?
    - Are the muscle cramps you experience painful?
    - Do your muscle cramps cause lasting pain in the area of the cramp?
    - Considering the muscle cramps you've had in the past month, how would you rate how painful your worst muscle cramp was on a scale from 0-10 with 0 being no pain and 10 being the worst pain you have ever experienced?
    - Again, considering the muscle cramps you've had in the past month, how would you rate how painful your average muscle cramp was on a scale from 0-10 with 0 being no pain and 10 being the worst pain you have ever experienced?
    - Overall, how would you rate the amount of bodily pain you have had in the past month because of muscle cramps on a scale of 0-10 with 0 being no pain and 10 being the worst pain you have ever experienced?
- Are muscle cramps in some places more painful than others?
- Are there other lasting effects of the muscle cramps? Stiffness? Weakness? Tiredness?

Amount of Bother

- Have the muscle cramps you've had in the past month bothered you? If so, in what ways?
    - [If needed] Do they have lasting effects (For example, does it change your plans for the rest of the day)? Does it affect your sleep? Does it affect your level of activity? Does it cause you to have anxiety?
- Are muscle cramps in some places more bothersome than others?
- Considering the muscle cramps you've had in the past month, how would you rate how bothersome they were on a scale from 0-10 with 0 being no bother at all and 10 being extremely disruptive to my life?

Pain Interference/Life Impact

- How do muscle cramps interfere with or get in the way of your life?
    - How does it impact:
        - what you do to take care of yourself every day?
        - what you eat? How much salt you eat? How much water you drink? Other parts of your diet?
        - how physically active you are?
        - your ability to work?

        - your mood?
        - how you interact with your family or friends?
        - how you feel about yourself?
    - What would you like to do that you can't do because of muscle cramping?
        - [*If needed*] Would you be more active? Would it cause less anxiety as you complete your dialysis treatment? Do you feel you would be more productive with your time?
    - Has muscle cramping interfered with your life more since COVID-19 crisis began or is your experience about the same as before COVID?

Treatment impact

- Does having muscle cramps affect how you prepare for or receive dialysis treatment?
    - Do you avoid doing certain things because you think they will bring on the cramps?
    - [APPLIES TO IN-CENTER HD AND HOME HD ONLY]: Do you skip or shorten dialysis sessions because of muscle cramps?
    - Do you do things differently to avoid getting cramps?
        - [If needed] Are there changes in what you eat or drink? Are there changes in the medications you take? Anything else?
- How, if at all has the COVID-19 crisis affected how you prepare for or receive dialysis treatment?
    - Do you think you are receiving the same level of attention as before from staff when you have muscle cramps?

Definition of muscle cramping for people on dialysis

- If you had to come up with a definition that describes what "muscle cramping for people on dialysis" is, what would you say?
    - Here's a possible definition: "Muscle Cramps are involuntary painful muscle contractions anywhere on the body, occurring during or between dialysis treatments, day or night."
        - Does this capture what you experience?
        - If not, what is missing?

Communication

- Who do you tell about your muscle cramps? (family, nurse, etc.)
    - Since the COVID-19 crisis, how have you told your health care providers about your muscle cramps? Telehealth platforms (apps, portals)?
- How often do you tell someone else about your muscle cramps?
    - Are you hesitant to tell others about your muscle cramps? If so, why?

- Does telling someone help? If yes, how does it help?

Remedies

- Have you heard of or used any treatments or remedies that help relieve muscle cramping? If so, what?
- What has your health care provider told you about muscle cramps and how to manage them?

Reactions to framework

- What is your overall reaction to what you see here? What do you think it trying to communicate? Is it effective in communicating this?
    - What stands out to you?
        - What do you like? Anything you dislike?
    - Is there anything unclear or confusing to you? Anything that might be confusing to others?
    - What are your thoughts on the "Changes in the way I act" circle?
        - Does this include the things that are most important? How so?
        - If not, what's missing?
        - Should anything be removed and replaced?
    - What are your thoughts on the "Changes in how I feel" circle?
        - Does this include the things that are most important? How so?
        - If not, what's missing?
        - Should anything be removed and replaced?
        - How is "I can't sleep" different from "I don't sleep well"?
    - What are your thoughts on the "Changes in things I can do" circle?
        - Does this include the things that are most important? How so?
        - If not, what's missing?
        - Should anything be removed and replaced?
- **PRIORITIZATION**:
    - Who thinks that the "Changes in the way I act" bubble is most important to address?
    - Who thinks that the "Changes in how I feel" bubble is most important?
    - Now, who thinks that the "Changes in things I can do" is most important?
    - For those who chose "Changes in the way I act", what did you think about when selecting that impact?
        - Looking within this circle, which two changes would you say are most important for patients?

    - For those who chose “Changes in how I feel”, what did you think about when selecting that impact?
        - Looking within this circle, which two changes would you say are most important for patients?
    - For those who chose “Changes in things I can do”, what did you think about when selecting that impact?
        - Looking within this circle, which two changes would you say are most important for patients?
- In your opinion, is this framework valuable? If so, in what ways? What would you change to make it more useful?
- How would you explain this framework to other patients and families?
